# Enhancement of Magneto-Induced Modulus by the Combination of Filler and Plasticizer Additives-Based Magnetorheological Elastomer

**DOI:** 10.3390/ma15186396

**Published:** 2022-09-15

**Authors:** Muntaz Hana Ahmad Khairi, Ervina Efzan Mhd Noor, Ubaidillah Ubaidillah, Siti Aishah Abdul Aziz, Saiful Amri Mazlan, Siti Maisarah Ahmad Tarmizi, Nur Azmah Nordin

**Affiliations:** 1 Engineering Materials and Structures (eMast) iKohza, Malaysian-Japan International Institute of Technology, Universiti Teknologi Malaysia, Jalan Sultan Yahya Petra, Kuala Lumpur 54100, Malaysia; 2 Faculty of Engineering and Technology, Multimedia University, Jalan Ayer Keroh Lama, Bukit Beruang, Melaka 75450, Malaysia; 3 Mechanical Engineering Department, Faculty of Engineering, Universitas Sebelas Maret, Jl. Ir. Sutami 36A, Kentingan Jebres, Surakarta 57126, Indonesia; 4 Faculty of Applied Sciences, Universiti Teknologi MARA Pahang, 26400 Bandar Tun Abdul Razak Jengka, Malaysia

**Keywords:** magnetorheological elastomer, magneto-induced modulus, CoFe_2_O_4_, plasticizer, solid additive

## Abstract

Filler additive is used to provide superior bonding in rubber matrix to enhance the storage modulus of magnetorheological elastomer (MRE). However, the magneto-induced modulus is reduced as the initial storage modulus increases. Therefore, this paper aims to increase the magneto-induced modulus and maintain the initial storage modulus by combining filler and plasticizer additives. Both types of additives have different functions, where cobalt ferrite (CoFe_2_O_4_) is capable of enhancing the maximum storage modulus and silicone oil (SO) reduces the initial storage modulus. Thus, four MRE samples have been fabricated using (a) no additive, (b) CoFe_2_O_4_, (c) SO, and (d) a combination of CoFe_2_O_4_ and SO. The sample’s hardness and magnetic properties were investigated via Durometer Shore A and Vibrating Sample Magnetometer (VSM), respectively. Furthermore, the rheological properties of MRE samples in terms of storage modulus were investigated upon the frequency and magnetic field sweep using a rheometer. The results demonstrated that the storage modulus of the MRE samples has increased with increasing the oscillation frequency from 0.1 to 50 Hz. Interestingly, the combination of additives has produced the largest value of magneto-induced modulus of 0.90 MPa as compared to other samples. Furthermore, their initial storage modulus was in between samples with SO (lowest) and without additive (highest). Therefore, fundamental knowledge in adding the combination of additives can offer solutions for a wide range of stiffness in MR device applications such as vibration and noise control devices, sensing devices, and actuators.

## 1. Introduction

Magnetorheological elastomer (MRE) is a smart material with tunable rheological properties influenced by an external magnetic field. It demonstrates reversible magnetic field-dependent modulus as well as damping properties. The micron-sized magnetic particles embedded in the elastomer or rubbery-like matrix materials are primarily responsible for MRE’s unique properties. Besides, MRE can be classified depending on the distribution of the magnetic particles, whether isotropic or anisotropic [1,2]. In isotropic MRE, the magnetic particles were uniformly dispersed in the matrix. Whilst in anisotropic MRE, the magnetic particles form a chain-like arrangement in the matrix because of the curing of MRE in the presence of a magnetic field [2]. Compared to isotropic MRE, anisotropic MRE has smaller gaps between the magnetic particles. Therefore, it can result in intense interactions between the particles, making them far more sensitive to increasing magnetic field strength, resulting in a significant change in stiffness. The difference in stiffness is called a magneto-induced modulus (or Magnetorheological (MR) effect) that becomes a crucial parameter to evaluate the performance of MRE.

Magnetic particles (either in isotropic or anisotropic MRE) play a significant role since this part directly interacts with the external magnetic field. The carbonyl iron particles (CIPs) are the most used magnetic particles in MRE fabrication. High CIPs loading in MRE up to 70 weight percent (wt.%) contributes to an increased storage modulus hence improving the magneto-induced modulus of MRE [3,4]. To further enhance the properties of MRE, the addition of small amounts of reinforcing filler, like metals, glasses, and carbon-based in the 0.1–15 wt.% range to MRE causes an improvement in the properties of the materials. The filler additives can enhance the magneto-induced modulus of MRE depending on their quality and quantity [1].

Carbon black (CB) is the most commonly used reinforcing filler in the rubber industry [5,6], and the influence of CB in MRE was studied by Chen et al. [7], Nayak et al. [8], and Fan et al. [9]. The experiments revealed that the MRE with CB obtained magneto-induced modulus up to 0.16 MPa. Furthermore, the presence of carbon black improved the physical properties of the given rubber compounds, such as tear strength and tensile strength. Moreover, according to Fan et al. [9], the different particle sizes of CB showed different interaction strengths with CIPs, thus altering the magneto-induced modulus. On the other hand, since 2011, carbon nanotubes (CNTs) have been reported to be widely used as filler additive materials in the fabrication of rubbers [10,11,12] and MRE [1,13,14,15,16] due to having the potential to produce excellent mechanical and rheological properties. For example, the experiment by Li and Sun [14] and Selvaraj et al. [16] revealed that MRE-containing CNTs enhanced the magneto-induced modulus up to 0.3 MPa compared to MRE without CNTs. Eventually, according to Aziz et al. [14], the magneto-induced modulus could be enhanced, even with a little amount of 0.1% wt. percentage of CNT. 

CNTs can outperform other carbon materials in terms of electrical and thermal properties. CNTs have various applications, including field emission, thermal conductors, energy storage, and structural materials. However, despite a decade of research, the full potential of using CNTs as reinforcements has been severely limited due to difficulties associated with entangled CNT dispersion during processing and poor interfacial interaction between CNTs and polymer matrices [11]. Besides CB and CNTs, there are several reports on using different filler additives in MRE, such as silicon carbide [17,18] and silica [19]. According to Wang et al. [17], utilization of silicon carbide improved the magneto-induced modulus by 2.1 times that in MRE without an additive. For the case of silica additive, Abd Rashid et al. [20] investigated the influence of silica in isotropic PDMS-based MRE. They found that the enhancement in the magneto-induced modulus of 0.33 MPa depended on the strength of the magnetic field and the percentage of the silica. All the mentioned approaches were intended to use the filler additive to enhance the MRE properties mainly by achieving the polymer–filler and filler–filler interactions [4,21].

Recently, ferrite spinel structures were gaining the interest of researchers due to their significant properties such as applicability at a higher frequency, high resistivity, more excellent heat resistance, and higher corrosion resistance. Cobalt ferrite (hereafter referred to as CoFe_2_O_4_) is one of the ferrites spinels that has become one of the magnificent candidates that has intrigued and proven to be a suitable additive in improving the composite [22] and the MR material [23,24]. Tarmizi et al. [23] discovered the benefit of CoFe_2_O_4_ in MR grease, and the results demonstrated that with the incorporation of 5 wt.% CoFe_2_O_4_ particles, the initial off-state viscosity was reduced by 86% compared to the pure sample, and improved the rheological properties upon applying the magnetic field. Accordingly, Aziz et al. [24] utilized modified CoFe_2_O_4_ in MRE, and the addition of the filler enhanced the magneto-induced modulus of 0.56 MPa compared to non-additive MRE, which was only 0.30 MPa, although CoFe_2_O_4_ is one of the remarkable fillers to use as an additive due to its multifunctional capabilities [23,24,25,26]. However, in most cases, the addition of filler increases the viscosity of the MRE, consequently reducing the magneto-induced modulus [27]. If this initial storage can be lowered despite the addition of filler, the resulting magneto-induced can be further enhanced. To encounter this issue, refinement of the MRE with a plasticizer [28,29,30,31] to be used together with filler (CoFe_2_O_4_) is necessary to lower the compound viscosity, hence reducing the initial storage modulus of MRE. 

The main technical contribution of this work is to obtain MRE with a high magneto-induced modulus of more than 0.56 MPa for widely industrial applications. To achieve an improved range of stiffness, the polymer−filler and filler−filler interactions must be improved by combining CoFe_2_O_4_ and SO plasticizer into the MRE. The plasticizer is used to reduce the initial storage modulus and improved the adhesiveness of the particles on the matrix surface. In contrast, the CoFe_2_O_4_ increases the maximum storage modulus by providing improved bonding with the rubber matrix, particularly at a high magnetic field. Thus, four different samples of MRE are investigated for their hardness and magnetic properties. Afterwards, the field-dependent dynamic viscoelastic properties are measured using a rheometer. Finally, the performance of MRE samples in terms of storage modulus and loss factor are compared and discussed.

## 2. Experimental Methods

### 2.1. Material Fabrication

This study was conducted using silicone rubber, type RTV-two NS625/Nippon Steel from Tokyo, Japan, as the MRE matrix. The magnetic particles used were carbonyl iron particles (CIPs) from BASF, Ludwigshafen, Germany, with an average size of 6 µm. The plasticizer additive used was silicone oil (SO), purchased from Nippon Steel, Tokyo, Japan. The densities of CIPs, silicone rubber and SO were 7.874, 1.08, and 1.26 g/cm^3^, respectively. The filler additives were cobalt ferrite (CoFe_2_O_4_) synthesized by our group [23] and had irregular shapes with a diameter of 1–3 µm. A group of anisotropic MRE samples was synthesized with two types of additives as per Table 1.

The CIPs in each sample was the same (70 wt.%). The materials were mixed thoroughly and uniformly using a mechanical stirrer (multi mix high speed dispersed (HSD), WiseStir HT-DX, PMI-Labortechnik GmbH, Lindau, Switzerland) for 10 min. Then, the stirred mixture of (silicone rubber, CIPs, and additive) mixed with catalysts as a crosslinking agent at 0.1 wt.% supplied from Nippon Steel Co., Tokyo, Japan. The mixture was then slowly poured onto the steel mold base of 1 mm in thickness and 70 mm in diameter. The mixture was spread evenly to ensure that the cured MRE had a good surface finish. The sample in the mold was kept curing for two hours with a magnetic field in terms of current applied from the electromagnetic circuit at room temperature for the solidification process. It is noted that all percentages used in the context refer to the weight percentage (wt.%).

### 2.2. Characterizations

Durometer Shore A (Sauter HBA 100-0, Freiburg, Germany) was used to determine the hardness of the MRE samples. The test was done five times for each sample to ensure that the results were consistent, and then the average value was calculated. A vibrating system magnetometer (Microsense, FCM-10, Lowell, MA, USA) was used to study magnetic hysteresis loops of MRE samples. The hysteresis loops for all MRE samples were measured up to 1100 kA/m of magnetic fields, and the test was carried out at room temperature. Then, a commercial rheometer (MCR 302 Anton Paar, Graz, Austria) equipped with an external magneto-controllable accessory, MRD 70/1T, was used to determine the rheological parameters of the MRE samples. The sample of 20 mm in diameter with 1 mm thickness was placed on a parallel plate. The measurements were conducted in the oscillatory mode under the shear frequency. The range was from 0.1 to 50 Hz, and the strain was kept constant at 0.01%. The applied currents were varied at 0, 1, 2, and 4 A during the test, which equates to 0, 200, 400, and 800 mT, respectively. Further, the magnetic flux density sweep test was conducted from 0 mt to 900 mT with constant strain at 0.01% and frequency at 1 Hz, to be used to calculate the magneto-induced modulus of each sample.

## 3. Results and Discussion

### 3.1. Hardness Test

Table 2 shows the measured hardness of each composition of MRE samples. Generally, all samples have different hardness values depending on with/out additives.

Sample 1 was the non-additive MRE, producing 62 Shore A and used as a benchmark in this assessment. For Sample 2, the hardness value increased from 62 to 68 Shore A. The increment was due to the rising metal filler (CoFe_2_O_4_) that acted as a reinforcing agent and consequently hardened the MRE matrix. The hardness results for sample 2 agreed well with several previous reports claiming that the hardness increased with the addition of the metal particles [32]. On the contrary, the addition of SO plasticizer reduced the hardness of Sample 3 to 54 shore A. The phenomenon occurred because the plasticizer has a specific solubility in rubber and contributed to the Brownian motion of polymer chains; therefore, it reduced the hardness of Sample 3. Meanwhile, for sample 4, the hardness value was in between Sample 2 and Sample 3, which was at 57 Shore A. The obtained result was expected, since Sample 4 possessed a combination of both CoFe_2_O_4_ and plasticizer.

### 3.2. Magnetic Properties

**Figure 1:**
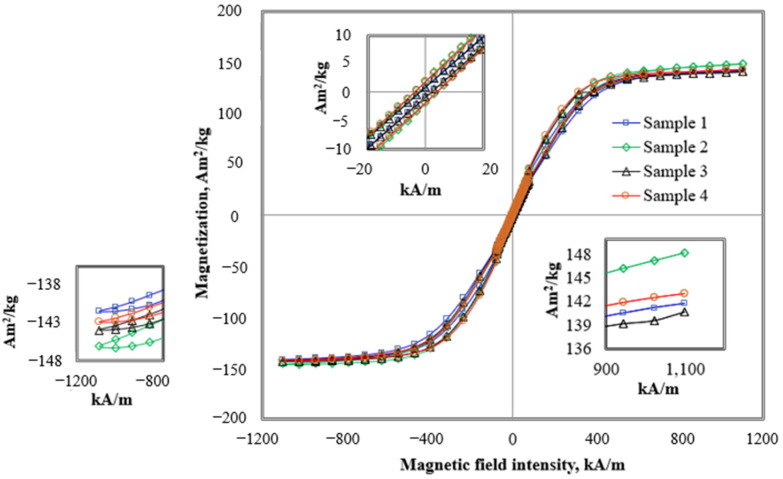
The magnetic properties of MRE samples.

The arrangement and orientation of magnetic particles under the influence of a magnetic field are known as magnetic moment and the magnetic properties of MRE samples with/out additives, as illustrated in Figure 1. The magnetic moment (also known as a magnetic dipole moment) is the strength of the response of a particle (in form of metal) to a magnetic field. It is a measure of the degree to which an object is affected by magnetism. Electrons and protons in quantum particles contributed to the magnetic moments that account for their magnetism due to the electrical charge and spin. From Figure 1, it has been observed that the magnetism type of the fabricated MRE is ferromagnetism, in which the ferromagnetic material exhibited a long-range ordering phenomenon at the atomic level, which causes the unpaired electron spins to line up parallel with each other in a region called a domain [33]. In addition, it was found that the hysteresis loops for all MRE samples were narrow with a high magnetic saturation (Ms), low remanent magnetization (Mr), and low coercive magnetic field (Hc). Thus, the fabricated MRE samples were considered soft magnetic materials.

The magnetic properties of MRE samples are shown in Table 3. The magnetic saturation value, Ms, for sample 1 was 141.71 Am^2^/kg, and the addition of CoFe_2_O_4_ to the matrix (Sample 2) has increased the Ms to 148.28 Am^2^/kg. The increment was resultant of the possession of 47.95 Am^2^/kg Ms value from micron-sized CoFe_2_O_4_ obtained from the previous study [23], which typically will support hundreds of magnetic domains to enhance the magnetization mechanisms [34]. Therefore, the existence of CoFe_2_O_4_ in the matrix enhanced the interaction between the particles, thus increasing the magnetic saturation due to the additional electrons and protons from CoFe_2_O_4_ that contributed to the magnetism as a result of the electrical charge and spinning [35].

In contrast, Sample 3 contained SO plasticizer, which is known to be a non-magnetic medium [28], and was used to lower the initial modulus and improve the adhesive properties of the CIPs on the matrix surface [36]. Therefore, the plasticizer layer that was formed on the surface of CIPs acted as a barrier that lessened the magnetization mechanisms, including domain wall motion and spin rotation. This phenomenon has reduced the magnetic saturation of sample 3 to 140.71 Am^2^/kg [34]. Meanwhile, Sample 4 contained both CoFe_2_O_4_ and SO plasticizer. The presence of CoFe_2_O_4_ modified the entire interaction inside the matrix, thus helping to improve the magnetic saturation. However, at the same, the presence of SO plasticizer in the sample restricted the magnetization, resulting in magnetic saturation up to 143.05 Am^2^/kg. Therefore, the magnetic saturation value of Sample 4 was in between Sample 2 and Sample 3.

On the other hand, the magnetic retentivity, Mr, refers to the amount of magnetization that remained in the MRE after the applied magnetic field was removed. It shows a low value (range between 0.88 to 1.95 Am^2^/kg) for all samples. It is noted that lower remanence is necessary for a specific application such as sensor devices, where the sensor material must respond quickly due to its sensitivity to the stimuli. Meanwhile, all samples exhibited different coercivities, Hc which refers to the measurement of the magnetizing force required to drive reverse magnetization once it has been saturated. The Hc for sample 1 was 1.96 kA/m, while for Sample 2, the Hc has increased to 3.91 kA/m. Meanwhile, the addition of SO plasticizer (Sample 3) insignificantly affected the Hc value, 1.98 kA/m, which was almost similar to Sample 1. It could be observed that in the presence of CoFe_2_O_4_ in Samples 2 and 4, the Hc has increased, however, the presence of SO plasticizer in Sample 4 has slightly reduced the Hc value. Thus, the Hc for Sample 4 was 3.88 kA/m.

### 3.3. Rheology Test-Magneto-Induced Modulus

**Figure 2:**
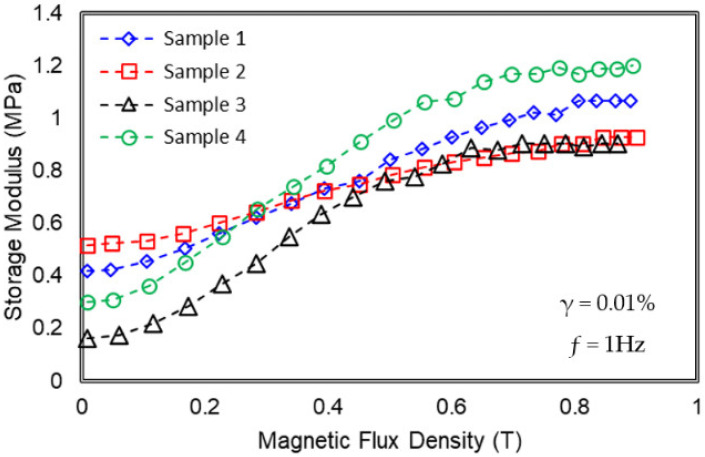
Storage modulus MRE samples at various magnetic flux densities.

The shear storage modulus (G′) of MRE under different magnetic flux densities is shown in Figure 2. The storage modulus of all samples showed an increasing pattern with the increasing magnetic flux density from 0 to 900 mT. Even though all the MRE samples have a fixed amount CIPs of 70 wt.%, it was interesting to see how those different types or the combination of the additives had affected the curves by showing different increased patterns from the initial to the maximum value with the increment of the magnetic field. Generally, the storage modulus in the absence of a magnetic field (initial storage modulus) was dependent on the modulus nature of the sample.

The initial storage modulus of Sample 2 was the highest and followed by Sample 1, Sample 4, and Sample 3. In most cases, the addition of filler additives has increased the storage modulus of the MRE, while the addition of plasticizers has reduced the initial storage modulus [37]. It is well-known that plasticizer contributes to the Brownian motion of polymer chains, and consequently reduces the viscosity of the rubber compound [38]. Therefore, to maintain the initial storage modulus of MRE, the filler (CoFe_2_O_4_) should be combined with a plasticizer (SO), as shown in Sample 4.

Meanwhile, for the on-state condition, generally, the stiffness (or modulus) of all MRE samples was changed, subjected to the interaction between magnetic particles due to the application of the magnetic field. The summary of rheological properties such as initial modulus *G*′_0_, maximum modulus *G*′*_max_*, and magneto-induced modulus Δ*G*′ MR effect of each sample are presented in Table 4. The following equation is used to compute the absolute or magneto-induced modulus MR effects of each sample:

(1) $$Magneto-induced\;modulus,\;\Delta G^{\prime }={G^{\prime }}_{max}-{G^{\prime }}_{0}$$

In Table 4, from the experimental results, the magneto-induced modulus of MRE in Sample 4 demonstrated the highest value as compared to the other samples; Sample 4 > Sample 3 > Sample 1 > Sample 2. The combination of CoFe_2_O_4_ and SO plasticizer in sample 4 has enhanced the interaction between the polymer–polymer and polymer–filler, thus resulting in a maximum modulus up to 1.2 MPa at a magnetic flux of 0.9 T, which at the same time provided the highest range of magneto-induced modulus. Nonetheless, Sample 2 showed the highest initial storage modulus representing a stiffer material, and as the magnetic flux density increased, the modulus also further increased. However, the modulus increment from its initial modulus was low due to the lessened interaction between particles, caused by the restriction of the stiff matrix as a result of the CoFe_2_O_4_ bonding within the matrix. For that reason, Sample 2 exhibited a low magneto-induced modulus of 0.41 MPa at 0.9 T. On the other hand, even though Sample 3 possessed the lowest initial modulus of 0.16 MPa, its magneto-induced was higher than Sample 2 due to the influence of SO plasticizer.

### 3.4. Rheology Test-Frequency Sweep

**Figure 3:**
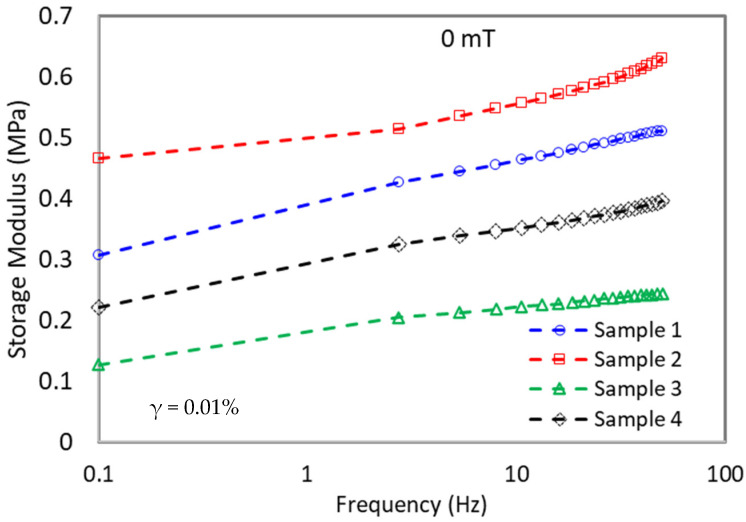
Storage modulus for MRE samples as a function of the frequency at off state.

The frequency sweep was performed within the linear viscoelastic range (γ_0_ = 0.01%) in the range of 0.1 to 50 Hz. The values of shear storage modulus G′ reflect the stiffness of a material, whereby it is defined as the ratio of stress to strain when the material is deformed. Therefore, the higher the modulus, the stiffer the material in which more stress is necessary to cause deformation. In this study, a frequency sweep test was conducted using various magnetic flux densities (0, 200, 400, and 600 mT) and is demonstrated in Figure 3. 

**Figure 4:**
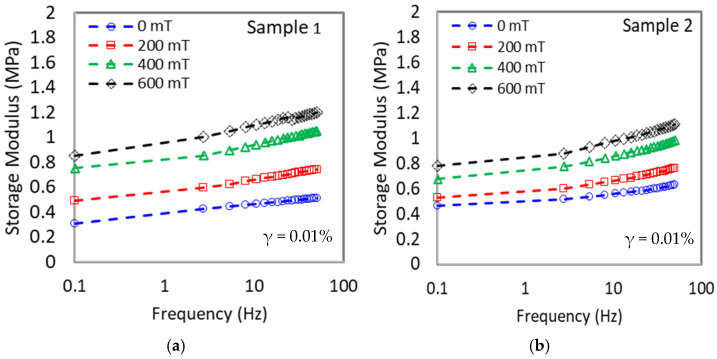

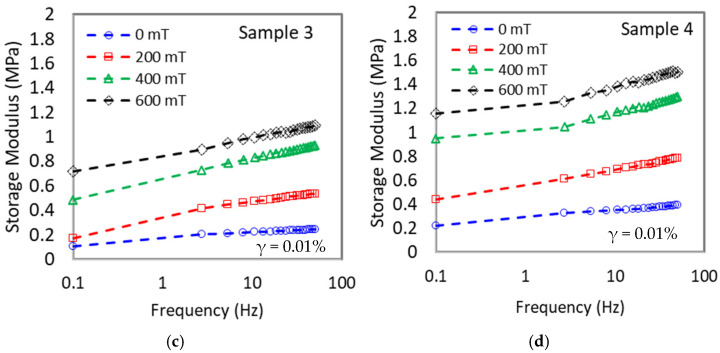
Variations of shear storage modulus with frequency at different magnetic flux densities for (**a**) Sample 1, (**b**) Sample 2, (**c**) Sample 3, and (**d**) Sample 4.

On the other hand, the initial storage modulus for all MRE samples increased parallel with the increment of frequency for every magnetic flux density as shown in Figure 4. This might be attributed to the vibration of CIPs in the presence of a magnetic field, which began to attract each other and caused the samples to become hardened hence increasing the initial storage modulus [39]. However, every sample has a different pattern of modulus increment at each magnetic flux density (0, 200, 400, and 600 mT). For example, as referred to in Table 5 (extracted from Figure 4a–d), when the shear storage moduli are measured at B = 0 and B = 600 mT, the highest magneto-induced modulus for sample 1 are 0.54 and 0.58 MPa at the frequency of 0.1 and 50 Hz, respectively. For all samples, the magneto-induced were higher at 50 Hz as compared to 0.1 Hz. This observable fact was as the frequency increased, the sample was hard to deform back to its original state as the chains of rubber molecules cannot be stretched hence further stiffening the MRE resulting in higher storage modulus [40]. It was noted that sample 1 was a non-additive MRE and used as a control sample in this study. 

Meanwhile, with the addition of CoFe_2_O_4_ to the matrix (Sample 2), the magneto-induced modulus decreased to 0.31 and 0.48 MPa at 0.1 and 50 Hz, respectively. The decrement was due to a stiffer matrix that restricted the particles’ attractions, thus lowering the magneto-induced modulus. Meanwhile, with the addition of SO plasticizer (Sample 3), the magneto-induced modulus has increased to 0.60 MPa (0.1 Hz) and 0.85 MPa (50 Hz). The softer matrix deregulates CIPs attraction when subjected to the magnetic field, hence increasing the magneto-induced modulus [41]. Remarkably, when CoFe_2_O_4_ and SO were added together in Sample 4, the magneto-induced modulus reached the highest value of 0.94 MPa. Moreover, the increment of magneto-induced modulus was enhanced further to 1.11 MPa at a high frequency of 50 Hz. The significant increment in the value was due to the dual effect of both CoFe_2_O_4_ and SO plasticizer. The addition of SO plasticizer has lowered the initial storage modulus, while the addition of CoFe_2_O_4_ has increased the final storage modulus by providing a stiffer matrix at a higher magnetic field, where the magneto-induced was more predominant at a higher frequency.

**Figure 5:**
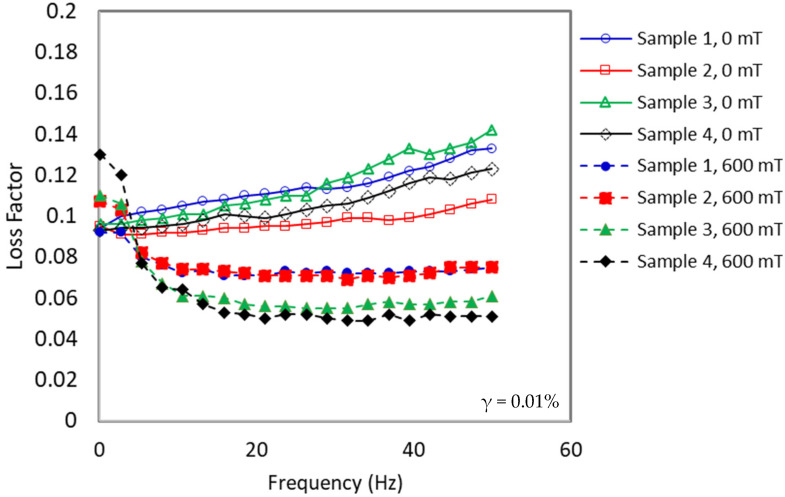
The loss factor of MRE samples as a function of the frequency at on- and off-state conditions.

The comparison of loss factor curves at off- and on-state conditions portrayed in Figure 5 represents the mechanical damping of the viscoelastic material, which is attributed to the ratio of dissipated energy stored to retained energy (tan δ = *G*″/*G*′) during the deformation of materials. According to Haining et al. [42], the loss factor manifested the difference in strength between the loss and storage moduli. The low loss factor value indicated that the behavior of the elasticity of the material was more prevalent than its viscous nature. 

It was apparent that the loss factor value at the on-state (B = 600 mT) condition was lower than that of the loss factor at the off-state (B = 0 mT) condition. In off-state conditions, the loss factor increased linearly by increasing the frequency. In contrast, under on-state conditions, the loss factor initially decreased rapidly at low frequency (below 20 Hz) and then the curves reached saturation values at a frequency higher than 20 Hz. The trend values for off-state and on-state conditions were different due to the different responses of both states to external stimuli. The stimulus in the off-state condition was from the influence of frequency, whereas the stimulus in the on-state condition was from a combination of magnetic field and the influence of frequency. 

In off-state conditions, the interaction of matrix and particles was relatively weak, thus easily producing sliding energy dissipation under the applied shear stress. Furthermore, the force generated between the particles would also increase the energy dissipation, which is caused by the interfacial sliding hence increasing the loss factor. The continuous interfacial sliding was further increased with increasing frequency due to typical shear-stiffened shear frequency [43]. However, a strong interaction force occurred between the particles and the rubber matrix, when a higher magnetic field was applied to the MRE sample under on-state conditions, resulting in decreasing in sliding displacement. While the shear-stiffened shear frequency phenomenon still occurred, the magnetic force was more dominant during the interaction, lowering the loss factor even further. On the other hand, at higher frequency (>20 Hz) under off-state conditions, the trend increased with increasing frequency due to the shear-stiffened shear frequency effect. However, at on-state conditions, a non-linear trend with fluctuating loss factor values was observed. Variabilities in polymer chain movement during particle–particle and matrix–particle interactions were associated with the energy released mechanism in the matrix network [44].

## 4. Conclusions

In this study, MRE samples using CoFe_2_O_4_, SO plasticizer, and a combination of them were successfully fabricated. The overall properties related to the hardness, magnetic, and rheological properties were experimentally investigated. Specific results are summarized as follows:

(a) MRE samples have different hardness values depending on the additive types. Sample 4, with a combination of CoFe_2_O_4_ and SO plasticizer, reduced the hardness of the MRE from 62 to 57 shore A compared to non-additive MRE.
(b) The magnetic saturation, retentivity, and coercivity were influenced by the addition of magnetic additive (CoFe_2_O_4_) because electrons and protons in quantum particles contributed to the magnetic moments that account for their magnetism as a result of their electrical charge and spin. In contrast, the addition of a non-magnetic additive (SO plasticizer) has reduced the magnetic properties due to no contributing to the magnetization activities.
(c) Sample 4 exhibited the highest magneto-induced modulus of 0.90 MPa, which was 38% higher than 0.65 MPa (Sample 1). The MRE with sole additive, Sample 2 and Sample 3, have obtained magneto-induced of 0.40 MPa and 0.75 MPa, respectively.

The resultant magneto-induced modulus of sample 4 was much larger due to the enhancement of the polymer–filler and filler–filler interactions by the combination of solid and plasticizer additives. Hence, the combination of additives (CoFe_2_O_4_ + SO plasticizer) can be beneficial in many industrial applications, especially in artificial muscle, soft actuators, and biomedical sensors.

## Figures and Tables

**Table 1:** MRE samples with additive contents.

Sample No.	Sample Name	Additive
1	MRE-Pure	None
2	MRE-CoFe_2_O_4_	5% CoFe_2_O_4_
3	MRE-SO	10% SO
4	MRE-(CoFe_2_O_4_ + SO)	5% CoFe_2_O_4_ and 10% SO

**Table 2:** MRE samples with additive contents.

No	Sample Name	Shore-A
1	MRE-Pure	62
2	MRE-CoFe_2_O_4_	68
3	MRE-SO	54
4	MRE (CoFe_2_O_4_ + SO)	57

**Table 3:** Magnetic properties of MRE samples with/out additives.

No	Samples	Magnetization, M_s_ (Am^2^/kg)	Retentivity, M_r_ (Am^2^/kg)	Coercivity, H_c_ (kA/m)
1.	MRE-Pure	141.71	0.88	1.96
2.	MRE-CoFe_2_O_4_	148.28	1.95	3.91
3.	MRE-SO	140.71	0.89	1.98
4.	MRE-(CoFe_2_O_4_ + SO)	143.05	1.85	3.88

**Table 4:** The rheological properties of MRE samples under the current sweep.

Sample No.	Sample Name	G_0_ [MPa]	G′_max_ [MPa]	Magneto-Induced, ∆G′ [MPa]
1	MRE-pure	0.42	1.07	0.65
2	MRE-CoFe_2_O_4_	0.52	0.93	0.41
3	MRE-SO	0.16	0.91	0.75
4	MRE-(CoFe_2_O_4_ + SO)	0.30	1.20	0.90

**Table 5:** The rheological properties of MRE samples under frequency sweep.

Sample No.	Sample Name	G_0_ [MPa] 0.1 Hz	G′_max_ (600 mT) [MPa] 0.1 Hz	Magneto-Induced, ∆G′ [MPa] 0.1 Hz	G_0_ [MPa] 50 Hz	G′_max_ (600 mT) [MPa] 50 Hz	Magneto-Induced, ∆G′ [MPa] 50 Hz
1	MRE-pure	0.31	0.85	0.54	0.51	1.09	0.58
2	MRE-CoFe_2_O_4_	0.47	0.78	0.31	0.63	1.11	0.48
3	MRE-SO	0.11	0.71	0.60	0.24	1.09	0.85
4	MRE-(CoFe_2_O_4_ + SO)	0.22	1.16	0.94	0.4	1.5	1.1
